# Gain-of-function mutant p53 promotes the oncogenic potential of head and neck squamous cell carcinoma cells by targeting the transcription factors FOXO3a and FOXM1

**DOI:** 10.1038/s41388-017-0032-z

**Published:** 2017-12-22

**Authors:** Noriaki Tanaka, Mei Zhao, Lin Tang, Ameeta A. Patel, Qing Xi, Hieu T. Van, Hideaki Takahashi, Abdullah A. Osman, Jiexin Zhang, Jing Wang, Jeffrey N. Myers, Ge Zhou

**Affiliations:** 1 Department of Head and Neck Surgery, The University of Texas MD Anderson Cancer Center, Houston, TX USA; 2 Department of Bioinformatics and Computational Biology, The University of Texas MD Anderson Cancer Center, Houston, TX USA

## Abstract

Many mutant p53 proteins exert oncogenic gain-of-function (GOF) properties that promote cancer cell invasive growth and metastasis, yet the mechanisms mediating these functions still largely remain elusive. We show here that overexpression of the GOF mutant p53 G245D and other GOF p53 mutants enhances the invasive cell growth of p53-deficient head and neck squamous cell carcinoma (HNSCC) UM-SCC-1 cells both in in vitro three-dimensional culture and in an in vivo orthotopic nude mouse model of HNSCC through a novel transcription-independent mechanism. We demonstrate that the expression of the oncogenic forkhead transcription factor *FOXM1* is upregulated by GOF mutant p53s. Moreover, we show that overexpression of GOF mutant p53 G245D decreases the AMP-activated protein kinase (AMPK)-mediated phosphorylation of FOXO3a, a tumor suppressive forkhead transcription factor, leading to its cytoplasmic accumulation. This downregulation of FOXO3a’s activity, in turn, leads to de-repression of *FOXM1* expression. Importantly, we show that either overexpression of *FOXO3a* or downregulation of *FOXM1* impairs both GOF mutant p53-mediated cell invasion in vitro and pulmonary metastases of UM-SCC-1 cells in vivo. Finally, not only do oral cancer patients with p53 mutations exhibit higher levels of *FOXM1* expression than patients with wild-type p53, but also HNSCC patients with *TP53* mutations and high levels of *FOXM1* expression have the poorest survival outcomes. Given our prior demonstration that GOF mutant p53s inhibit AMPK, our current study, establishes and demonstrates a novel transcription-independent GOF mutant p53-AMPK-FOXO3a-FOXM1 signaling cascade that plays an important role in mediating mutant p53s’ gain-of-function activities in HNSCCs.

## Introduction

Mutations of the *TP53* tumor suppressor gene are the most frequent of all somatic genomic alterations in head and neck squamous cell carcinomas (HNSCCs), with a mutation frequency in non-human papilloma virus-associated HNSCC cases ranging from 75 to 85% [1–3]. Clinically, *TP53* mutations are significantly associated with shorter survival time and tumor resistance to radiotherapy and chemotherapy in HNSCC patients [4–6]. Some p53 mutations are associated with gain-of-function (GOF) activities that can enhance tumor progression, metastatic potential, and/or drug resistance when overexpressed in cells lacking wild-type *TP53* [7–9]. However, the mechanisms involved in mutant p53 GOF activities still remain largely unclear.

Although mutant p53s usually cannot directly regulate the expression of the wild-type p53’s target genes, studies have found that the mutants can activate other genes by binding to promoters [8], cooperate with transcription factors to affect target gene expression [8, 10, 11], and can also participate in epigenetic gene regulation [12, 13]. Furthermore, it has been previously found that cytoplasmic GOF mutant p53s can regulate oncogenic activities through transcription-independent mechanisms [14–16]. Specifically, we have shown that inhibition of AMP-activated protein kinase (AMPK), a master energy sensor, is one mechanism through which mutant p53s achieve GOF activities in HNSCC cells [16].

FOXM1 and FOXO3a belong to the forkhead box superfamily proteins [17]. FOXM1, a member of the FOXM subfamily of transcription factors that has three isoforms, FOXM1a, -b, and -c [18], is highly expressed in various carcinomas, including cancers of the liver, prostate, brain, breast, lung, colon, pancreas, skin, cervix, ovary, blood, nervous system, oral cavity, and head and neck [19, 20]. Studies have shown that FOXM1, an oncogenic transcription factor, plays a variety of roles in promoting processes such as cell cycle progression, DNA repair, angiogenesis, stemness, tumor cell migration, invasion, and metastasis, contributing to tumor initiation, progression, and drug resistance through different mechanisms [17,19–21]. In contrast, FOXO3a, a member of the FOXO subfamily of transcription factors, is generally known as a tumor suppressor that plays roles in cell cycle arrest, DNA repair, hypoxia response, aging, longevity, differentiation, stress resistance, metabolism, apoptosis, and inhibition of cell invasion and metastasis [17, 22–24].

Both FOXM1 and FOXO3a are subjected to transcriptional and post-translational regulation. While FOXM1 is transcriptionally regulated by transcription factors, such as E2F, ER, and FOXO family members, and is phosphorylated by cyclin-CDK, PLK, CHK2, p38, and ERK [17–19], FOXO3a is known to be posttranslationally modified by acetylation, ubiquitylation, methylation, O-GlcNAcylation, and phosphorylation by kinases such as AKT, ERK, IKKβ, MST1, p38, and AMPK [17, 23]. Among these kinases, AKT, ERK, and IKKβ promote FOXO3a’s cytoplasmic retention and inactivate its function [25–27], whereas p38, MST1, and AMPK promote FOXO3a’s nuclear localization and activate its function as a transcription factor [23, 28–30]. More importantly, FOXO3a transcriptionally antagonizes *FOXM1* expression through different mechanisms, including direct transcriptional repression of *FOXM1* that leads to sustained inhibition of *FOXM1* gene expression [17, 19, 31, 32].

Previously, we showed that inhibition of AMPK, a master energy sensor and metabolic regulator, is one of the mechanisms through which mutant p53s achieve GOF activities in HNSCC cells [16]. To further study the GOF mechanisms of mutant p53, we have used isogenic HNSCC cell lines expressing GOF mutant p53s. We found that *FOXM1* expression is upregulated by GOF mutant p53s. We further demonstrated that GOF mutant p53s inhibit AMPK-mediated phosphorylation and nuclear localization of FOXO3a with a concomitant loss of FOXO3a’s suppression on *FOXM1* expression. Furthermore, we also showed both in vitro and in vivo that FOXO3a and FOXM1 are implicated in regulation of GOF mutant p53-mediated cell invasion and metastasis. Altogether, our study demonstrates that mutant p53s can gain oncogenic activities through a novel mechanism of modulation of the AMPK–FOXO3a–FOXM1 signaling axis in HNSCC cells.

## Results

### Identification of *FOXM1* as an up-regulated gene by expression of GOF mutant p53 G245D in UM-SCC-1 cells upon metabolic stress

**Fig. 1:**
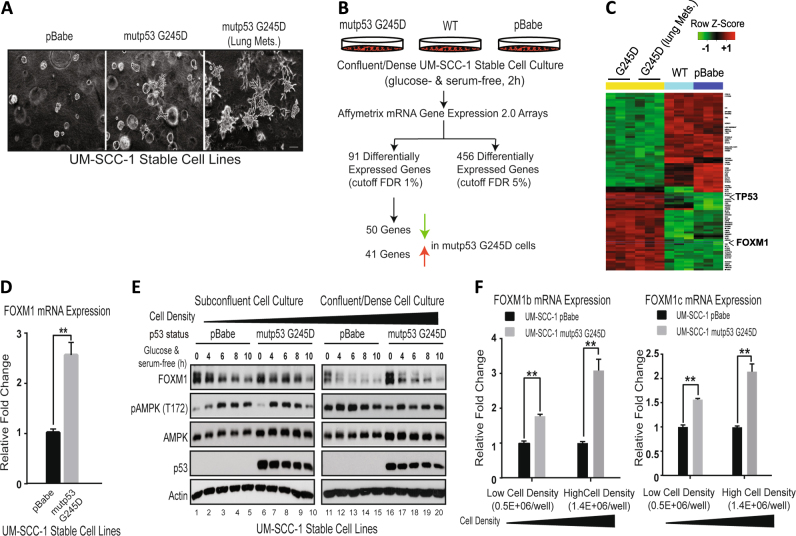
Identification of FOXM1 as an up-regulated gene by expression of GOF mutant p53 G245D in UM-SCC-1 cells. **a** GOF of mutant p53 (mutp53) G245D in UM-SCC-1 cells. Representative images of isogenic UM-SCC-1 stable cell lines grown in 3D culture. Cells labeled with Lung Mets., were cells derived from lung metastatic lesions after tail vein injection of UM-SCC-1 cells expressing G245D mutant p53. Bar, 200 μm. **b** Schematic diagram of experimental procedure for mRNA expression array analysis. *WT* wild-type, *FDR* false discovery rate. **c** Heatmap of 91 differentially expressed genes in mutant p53 G245D UM-SCC-1 cells *vs* the controls. **d** Real-time RT-qPCR analysis of *FOXM1* mRNA transcript from UM-SCC-1 stable cells 2 h after metabolic stress under confluent culture condition. **e** Western blot analysis of isogenic UM-SCC-1 stable cell lines in the presence or absence of metabolic stress under different cell-density culture conditions. **f** Real-time RT-qPCR analysis of mRNA transcripts of *FOXM1b* and *-1c* from UM-SCC-1 stable cells under different cell-density culture conditions. **p < 0.01

To study GOF mechanisms of mutant p53s, we first used HNSCC UM-SCC-1 cells that do not express endogenous p53 due to a splice-site mutation (hg19:chr17:7578370C > T) in the *TP53* gene and established isogenic cell lines expressing G245D, R175H, and C238F mutant p53s. As we described previously, when these cell lines were injected into the tail veins of nude mice, they caused a higher incidence of lung metastases than did control UM-SCC-1 cells without mutant p53 expression [9]. We harvested lungs from mice in which mutant p53 G245D UM-SCC-1 cells had been injected through the tail veins and developed a cell line from a lung metastatic lesion. We subsequently found that both the parental and lung metastatic lesion-derived mutant p53 G245D UM-SCC-1 cell lines exhibited a much more aggressive growth phenotype in in vitro three-dimensional (3D) collagen culture than did the control cells; the aggressive phenotype was characterized by the formation of tumor spheroids with numerous protrusions invading into the surrounding collagen matrix (Fig. 1a), supporting the GOF activities of G245D mutant p53 in these cells.

To further explore potential GOF mechanisms involved, we performed mRNA expression array analysis using RNA samples from over-confluent pBabe, wild-type p53, and parental and metastatic lesion-derived G245D mutant p53 UM-SCC-1 cells that were subjected to metabolic stress (glucose-free and serum-free culture conditions) for 2 h (Fig. 1b). Using a 1% cutoff false discovery rate, our analyses showed that 91 genes, including 50 down-regulated and 41 up-regulated genes, were differentially expressed in both parental and metastatic G245D UM-SCC-1 cells, when compared to wild-type and pBabe control cells (Fig. 1b, c) (Supplementary Table 1). Ingenuity Pathway Analysis (IPA; Qiagen) indicated that many of the top signaling pathways of the differentially expressed genes are involved in lipid metabolism, such as cholesterol biosynthesis and acetyl-CoA-biosynthesis, as well as the previously reported mevalonate pathway (Supplementary Fig. 1) [33]. Notably, among those differentially expressed genes, *FOXM1* is one of two transcription factors that was identified (Fig. 1c) and confirmed (Fig. 1d) to be up-regulated in G245D mutant p53 cells. Given the pleiotropic effects and oncogenic properties of the transcription factor FOXM1 [16, 17, 21], we next focused our investigations on the regulation of *FOXM1* by GOF mutant p53.

To validate the mRNA microarray results, we next performed Western blotting analysis. As shown in Fig. 1e, no obvious differences in FOXM1 levels were observed between the control and mutant p53 G245D UM-SCC-1 cells, when cells were cultured under a sub-confluent condition. However, when cultured under a high-cell-density condition, mutant p53 G245D UM-SCC-1 cells exhibited much higher levels of FOXM1 expression than the control cells. In addition, RT-qPCR analyses confirmed that the relative mRNA levels of *FOXM1b* and *FOXM1c* isoforms were up-regulated in G245D UM-SCC-1 cells in a cell density-dependent manner, in which a higher cell density was associated with higher levels of *FOXM1-b* and *-c* mRNA in G245D cells when compared to the control (Fig. 1f). Altogether, our results suggest that GOF mutant p53 up-regulates *FOXM1* gene expression in UM-SCC-1 cells, particularly under high-density cell culture conditions.

### GOF mutant p53s promote *FOXM1* expression both in vitro and in vivo

**Fig. 2:**
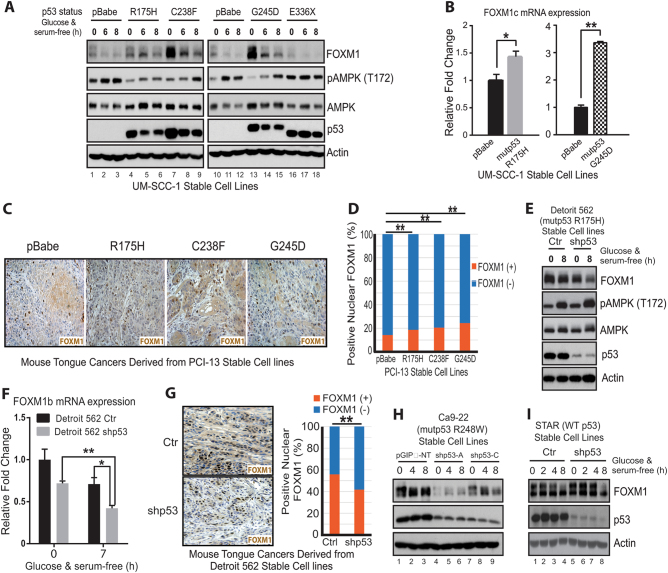
GOF mutant p53s G245D, C238F, and R175H promote *FOXM1* expression both in vitro and in vivo. **a** Western blot analysis of isogenic UM-SCC-1 stable cell lines in the presence or absence of metabolic stress under the high-density confluent culture condition. **b** Real-time RT-qPCR analysis of mRNA transcripts of *FOXM1c* from UM-SCC-1 stable cells under the confluent high-density culture condition. * < 0.05; **p < 0.01. **c** Representative images of FOXM1 IHC staining of tongue tumors derived from injection of isogenic PCI-13 stable cell lines. **d** Quantitation of nuclear FOXM1 staining of **c**. **p < 0.01. **e**,** h**–**i** Western blot analyses of Detroit 562, Ca9-22 and STAR stable cell lines in the presence or absence of metabolic stress under the confluent high-density culture condition. pGIPz-NT, non-target shRNA control. **f** Real-time RT-qPCR analysis of mRNA transcripts of *FOXM1b* from Detroit 562 stable cells under the confluent high-density culture condition. * < 0.05; ** < 0.01. **g** left, Representative images of IHC staining of FOXM1 in mouse tongue tumors derived from Detroit 562 stable cell lines; right, Quantitation of nuclear FOXM1 staining. **p < 0.01

Further analyses also indicated that GOF mutant p53s in addition to G245D can up-regulate *FOXM1* expression. For instance, mutant p53 C238F, which we previously had shown to have strong GOF activity both in vitro and in vivo [6, 9], strongly up-regulated FOXM1 in UM-SCC-1 cells (Fig. 2a). Moreover, R175H, although it has a weaker impact, also increased FOXM1 protein (Fig. 2a) and mRNA expression (Fig. 2b) in UM-SCC-1 cells. In contrast, E336X, a mutant with no GOF activity [16], failed to increase FOXM1 expression in UM-SCC-1 cells (Fig. 2a). Moreover, immunohistochemical (IHC) staining of tumors from an orthotopic implantation of PCI-13 (another HNSCC cell line that does not endogenously express *TP53*) stable cell lines expressing different mutant p53s grown in the tongues of nude mice [6] also showed that the ratio of positive nuclear staining of FOXM1 was significantly higher in the cell lines containing the mutant p53s R175H, C238F, and G245D than in the pBabe control cell line (Fig. 2c, d), indicating that FOXM1 expression is up-regulated by these GOF mutant p53s in vivo. In further support of the regulatory role of GOF mutant p53s on *FOXM1* expression, both FOXM1 protein and *FOXM1b* mRNA were decreased when mutant p53 was knocked down by shp53 in Detroit 562 cells, which endogenously express R175H mutant p53 (Fig. 2e, f). Consistent with that finding was the IHC staining data showing a much stronger positive nuclear staining of FOXM1 in tumor samples generated from control Detroit 562 cells than from shp53 Detroit 562 cells (Fig. 2g). Similarly, when mutant p53 R248W that showed a strong GOF activity in Ca9-22 cells (Supplementary Fig. 2a, b) was knocked down by p53 shRNA, FOXM1 protein levels were also significantly decreased (Fig. 2h). Finally, instead of down-regulation of *FOXM1* expression as observed in Detroit 562 and Ca9-22 cells (Fig. 2e–h), decreasing the p53 level by p53 shRNA in STAR cells, which contain endogenous wild-type p53, increased the levels of FOXM1 (Fig. 2i), suggesting that wild-type p53 inhibits FOXM1 expression.

Taken together, our results demonstrate that in contrast to wild-type p53, GOF mutant p53s up-regulate *FOXM1* expression both in vitro and in vivo in HNSCC cells.

### *FOXM1* expression is inversely correlated with and negatively regulated by AMPK activation in a cell density-dependent manner

Our results clearly showed that while levels of FOXM1 in UM-SCC-1 cells were suppressed by increased cell density (Supplementary Fig. 2c), expression of GOF mutant p53 in UM-SCC-1 cells was associated with increased *FOXM1* expression in a cell density-dependent manner (see above) (Fig. 1e, f). We previously observed that GOF mutant p53-mediated inhibition of AMPK activation is also dependent on cell density [16]. Interestingly, levels of *FOXM1* expression appeared to be inversely correlated with T172 phosphorylation of AMPK and further decreased after metabolic stress that induced AMPK activation (increased T172 phosphorylation) (Figs. 1e and 2a, e). Given our observations that GOF mutant p53s inhibit AMPK [16] but promote *FOXM1* expression (Figs. 1 and 2), the inverse correlation of FOXM1 expression and AMPK activation observed here (Figs. 1e and 2a, e) suggests that AMPK may be able to regulate FOXM1 expression in HNSCC cells.

**Fig. 3:**
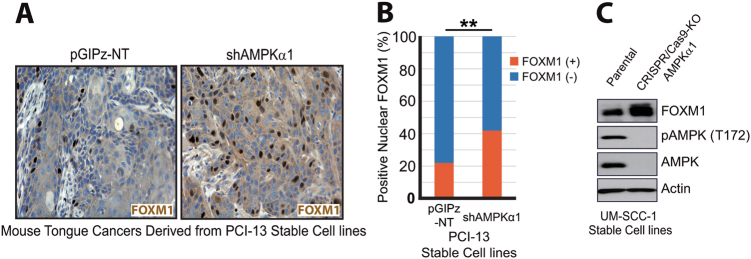
AMPK suppresses FOXM1 expression in HNSCC cells. **a** Representative images of FOXM1 IHC staining in mouse tongue tumors derived from PCI-13 stable cell lines. **b** Quantitation of nuclear FOXM1 staining of **a**. **p < 0.01. **c** Western blot analysis of CRISPR-Cas9/AMPKα1 KO UM-SCC-1 cell lines

To test this, we performed IHC staining using mouse tongue tumor samples derived from injections of the control and AMPKα1 knockdown (shAMPKα1) PCI-13 stable cell lines [16]. As shown in Fig. 3a, b, higher levels of FOXM1 positive staining were found in PCI-13 shAMPKα1 tumor samples than in the control PCI-13 tumors. To further investigate whether AMPK can regulate FOXM1 expression, we generated an AMPKα1 KO UM-SCC-1 cell line using the CRISPR/Cas9 system. Consistent with the above observation, the level of FOXM1 expression in AMPKα1 KO cells was higher than that in parental UM-SCC-1 cells (Fig. 3c). These results strongly suggest that AMPK can negatively regulate FOXM1 expression both in vitro and in vivo.

### FOXO3a, a downstream target of AMPK, inhibits GOF mutant p53-mediated *FOXM1* expression

**Fig. 4:**
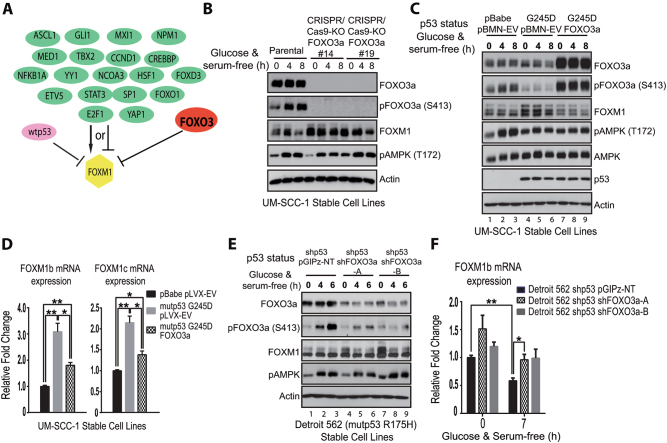
FOXO3a, a downstream target of AMPK, inhibits GOF mutant p53-mediated *FOXM1* expression in HNSCC cells. **a** Ingenuity Pathway Analysis of possible upstream transcription factors regulating *FOXM1* expression. **b** Western blot analysis of CRISPR-Cas9/FOXO3a KO UM-SCC-1 cell lines in the presence or absence of metabolic stress under the confluent high-density culture condition. **c** Western blot analysis of isogenic UM-SCC-1 stable cell lines in the presence or absence of metabolic stress under the confluent high-density culture condition. pBMN-EV, empty vector. **d** Real-time RT-qPCR analysis of mRNA transcripts of *FOXM1b* and *-1c* from UM-SCC-1 stable cells under the confluent high-density culture condition. * <0.05; **p < 0.01. pLVX-EV, empty vector. **e** Western blot analysis of Detroit 562 stable cell lines in the presence or absence of metabolic stress under the confluent high-density culture condition. **f** Real-time RT-qPCR analysis of *FOXM1b* mRNA transcripts from Detroit 562 stable cells in the presence or absence of metabolic stress under the confluent high-density culture condition. *<0.05; **p < 0.01

To identify potential mechanisms involved in AMPK-mediated regulation of FOXM1, we next examined potential upstream signaling of *FOXM1* using IPA, which summarizes previously reported upstream transcription factors that are able to modulate *FOXM1* expression directly or indirectly (Fig. 4a). Among those factors, FOXO3a can bind to the upstream promoter and suppress the transcription of *FOXM1* [31]. Most importantly, FOXO3a is a direct target of AMPK, and AMPK-mediated phosphorylation of FOXO3a can lead to the nuclear localization and activation of FOXO3a [23, 30]. Consistent with this, in AMPKα1 KO UM-SCC-1 cells, we observed decreased levels of FOXO3a phosphorylation (Supplementary Fig. 3a). All these factors led us to hypothesize that FOXO3a could be an important factor linking GOF mutant p53-mediated inhibitory effects on AMPK with *FOXM1* expression in UM-SCC-1 cells.

To test this possibility, we used the CRISPR/Cas9 technology to knock out FOXO3a in UM-SCC-1 cells. When compared to levels in the control, FOXM1 levels were significantly increased in the *FOXO3a* KO cells (Fig. 4b), indicating that FOXO3a has an inhibitory effect on *FOXM1* expression. Consistent with this, when we overexpressed *FOXO3a* in mutant p53 G245D-bearing UM-SCC-1 cells, mutant p53-mediated up-regulation of FOXM1 protein and mRNA levels were significantly diminished (Fig. 4c, d, Supplementary Fig. 3b). Similarly, although we showed that mutant p53 R175H knockdown in Detroit 562 cells resulted in decreased levels of FOXM1 expression (Fig. 2e, f), further knocking down FOXO3a in these cells partially restored the levels of FOXM1 protein and mRNA expression (Fig. 4e, f). All these results, taken together, strongly support that FOXO3a inhibits GOF mutant p53-mediated *FOXM1* expression in UM-SCC-1 and Detroit 562 cells.

### GOF mutant p53 inhibits AMPK-mediated phosphorylation and nuclear localization of FOXO3a

**Fig. 5:**
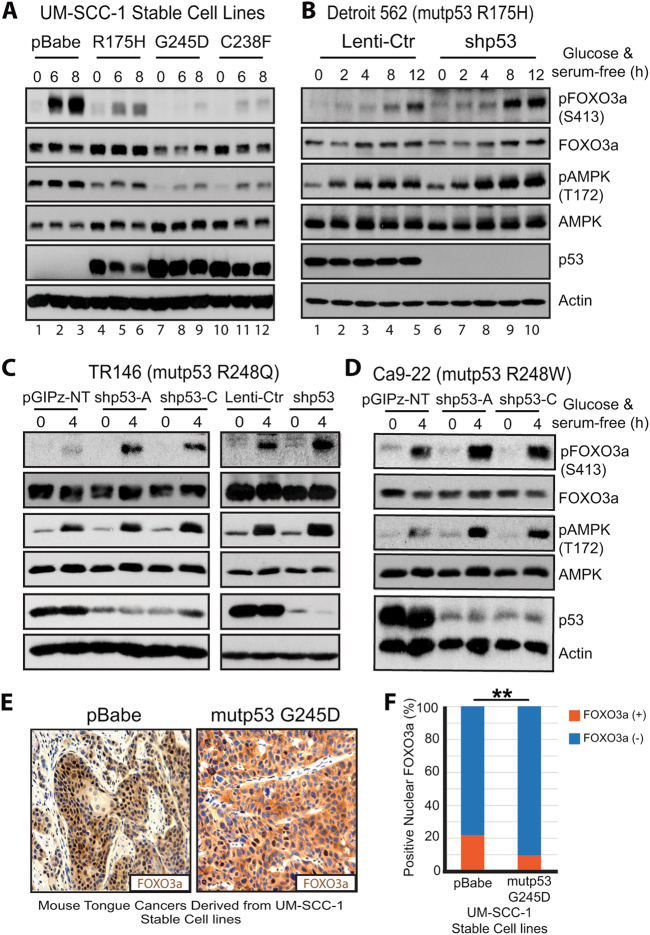
GOF mutant p53 inhibits AMPK-mediated phosphorylation and nuclear localization of FOXO3a. **a**–**d** Western blot analyses of isogenic UM-SCC-1, Detroit 562, TR146 and Ca9-22 stable cell lines in the presence or absence of metabolic stress under the confluent high-density culture condition. **e** Representative images of IHC staining of FOXO3a in mouse tongue tumors derived from UM-SCC-1 stable cell lines. **f** Quantitation of FOXO3a nuclear staining of **e**. **p < 0.01

To further investigate the role of GOF mutant p53 on FOXO3a activation, we examined the impact of mutant p53s on phosphorylation of FOXO3a at S413, one of six putative AMPK phosphorylation sites in FOXO3a [30]. Our results showed that while GOF mutant p53s (e.g., G245D and C238F) inhibited activation of AMPK induced by metabolic stress, they also significantly suppressed the S413 phosphorylation of FOXO3a that was induced by AMPK activation (Figs. 4c and 5a). In contrast, in Detroit 562, TR146 and Ca9-22 cells when mutant p53 R175H, R248Q and R248W were knocked down, respectively, which led to a higher level of metabolic stress-induced AMPK activation/phosphorylation, increased FOXO3a S413 phosphorylation was observed (Fig. 5b–d). Interestingly, while it exhibited a minimal impact on cell growth in 3D collagen culture (Supplementary Fig. 3c, e), knocking down mutant p53 in MDA1586 (R273L), PCI-15B (R273C), and UMSCC-17B (R273C) cells had no significant effect on AMPK activation and FOXO3a phosphorylation (Supplementary Fig. 3f–h). These results together strongly suggest that not all the mutant p53s are the same in their GOF activities and mechanisms.

Finally, IHC staining of oral tumor samples derived from UM-SCC-1 with mutant p53 G245D showed a higher portion of cytoplasmic staining of FOXO3a, whereas most FOXO3a in the control tumors was localized in the nucleus (Fig. 5e, f). Given that activated transcription factor FOXO3a functions in the nucleus [23], our findings that mutant p53 G245D inhibited phosphorylation and nuclear localization of FOXO3a (Fig. 5) support that GOF mutant p53 inhibits FOXO3a function.

### FOXO3a and FOXM1 modulate GOF mutant p53-mediated UM-SCC-1 cell invasion in vitro

**Fig. 6:**
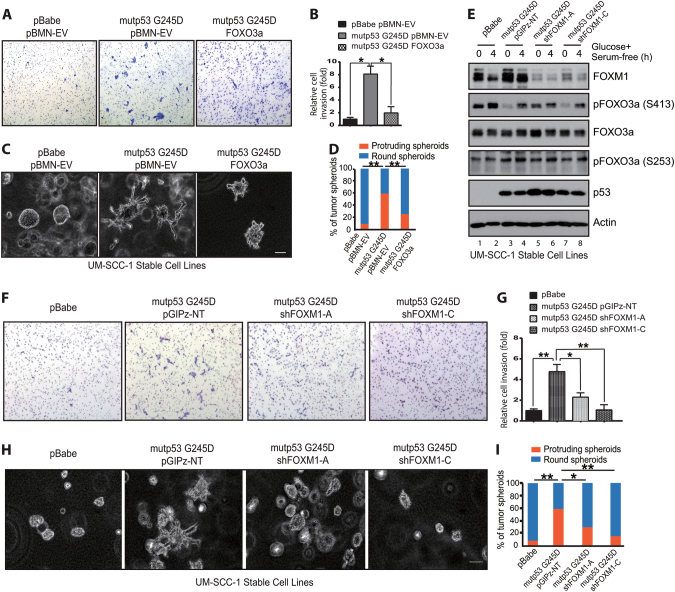
FOXO3a and FOXM1 modulates GOF mutant p53-mediated cell invasion in vitro. **a**, **f** Representative images from the Transwell invasion assay of UM-SCC-1 stable cell lines. **b**, **g** Quantitation of invasive cells in **a** and **f**, respectively. *p < 0.05; **p < 0.01. **c**, **h** Representative images of 3D cultures of UM-SCC-1 stable cell lines. Bar, 100 μm. **d**, **i** Quantitation of 3D tumor spheroids that were round or had protrusion in **c** and **h**, respectively. *<0.05; **p < 0.01. **e** Western blot analysis of isogenic UM-SCC-1 stable cell lines in the presence or absence of metabolic stress under the confluent high-density culture condition

Our results thus far (Figs. 1–5) strongly suggest that inhibition of AMPK-mediated FOXO3a activation could be an important mechanism contributing to GOF mutant p53-mediated *FOXM1* up-regulation in HNSCC cells. To further confirm the potential role of FOXO3a in mutant p53-mediated GOF phenotype, we performed an in vitro Transwell invasion assay and 3D cell cultures. In the invasion assay, overexpressing FOXO3a in UM-SCC-1 mutant p53 G245D cell line (Fig. 4c) significantly decreased the number of invasive cells compared to the vector control cell line (Fig. 6a, b). Similarly, reduced invasion was also observed in UM-SCC-1 mutant p53 R175H cells when FOXO3a was expressed (Supplementary Fig. 4a, b). In further support of FOXO3a’s role, in a 3D cell culture, G245D mutant p53 cells exhibited a more infiltrative phenotype with projecting protrusions, whereas the vector control UM-SCC-1 cells mainly formed round-shaped tumor spheroids (Fig. 1a); the control phenotype was further suppressed by overexpressing *FOXO3a*, leading to reduced formation of projecting protrusions (Fig. 6c, d).

To investigate the possible role of FOXM1 in mutant p53-mediated GOF activities, we used *FOXM1* shRNAs to stably knock down *FOXM1* expression in G245D (Fig. 6e) and R175H (Supplementary Fig. 5a) mutant p53 UM-SCC-1 cells. As a result, knocking down FOXM1 not only suppressed the mutant p53-mediated cell invasion across Matrigel-coated Transwells (Fig. 6f, g, Supplementary Fig. 5b, c) but also partially reduced the formation of projecting protrusions in UM-SCC-1 cells grown in 3D cultures (Fig. 6h, i).

These results demonstrated that both FOXO3a and FOXM1 can modulate mutant p53’s GOF properties, such that FOXO3a inhibits but FOXM1 promotes mutant p53-mediated cell invasion in vitro in UM-SCC-1 cells.

### FOXO3a inhibits but FOXM1 promotes GOF mutant p53-mediated pulmonary metastasis of UM-SCC-1 cells in vivo

**Fig. 7:**
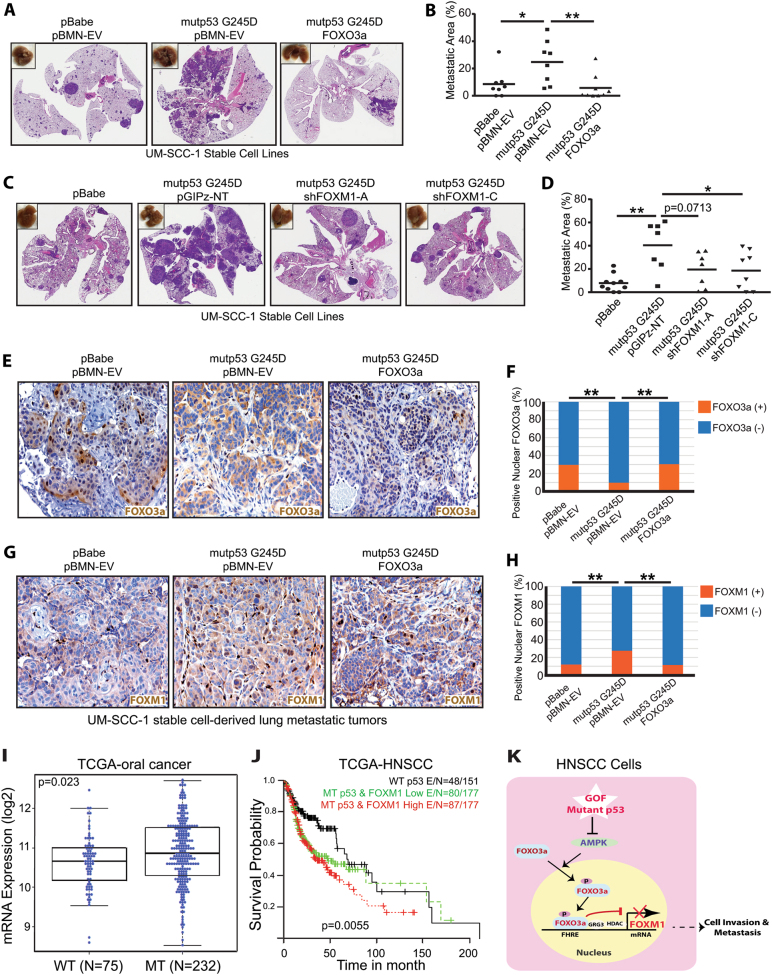
FOXO3a inhibits but FOXM1 promotes GOF mutant p53-mediated pulmonary metastasis of UM-SCC-1 cells in vivo. **a**, **c** Representative images of H&E-stained sections of lungs 13 **a** and 14 **c** weeks, respectively, after tail-vein injection of UM-SCC-1 stable cell lines. **b**, **d** Quantitation of the lung metastatic areas in **a** and **c**, respectively. *<0.05; **p < 0.01. **e**, **g** Representative images of FOXO3a **e** and FOXM1 **g** IHC staining of pulmonary metastatic lesions derived from the tail-vein injected UM-SCC-1 stable cell lines. **f**, **h** Quantitation of the positive nuclear staining of FOXO3 and FOXM1 in **e** and **g**, respectively. **p < 0.01. **i** *FOXM1* mRNA expression of oral cancers (*n* = 307) with wild-type (WT) or mutant (MT) *TP53* in the HNSCC TCGA dataset. 307 oral cancer samples were identified in 505 HNSCC TCGA tumor samples as tumors originating from subsites including oral cavity, oral tongue, floor of mouth, alveolar ridge, buccal mucosa, hard palate, and lip. **j** Survival of HNSCC patients with wild-type *TP53* (WT) (*n* = 151), mutant *TP53* (MT) and high levels or low levels of *FOXM1* expression (*n* = 354). High or low *FOXM1* expression was defined as the levels of the expression that are greater than (high) or less than (low) the median/mean expression in mutant p53 group. **k** Diagram of the working model of mutant p53 GOF in HNSCCs, in which GOF mutant p53s promote cell invasion and metastasis through regulation of the AMPK-FOXO3a-FOXM1 signaling axis

To further examine the role of FOXO3a and FOXM1 in vivo, we first used an orthotopic nude mouse model of oral cancer, in which UM-SCC-1 stable cell lines were injected into the tongues of nude mice. As shown in Supplementary Fig. 6a, b, whereas overexpression of mutant p53 G245D greatly promoted the tumor growth when compared to the control cells, overexpression of FOXO3a (Supplementary Fig. 6a) or downregulation of FOXM1 (Supplementary Fig. 6b) in mutant p53 G245D UM-SCC-1 cells showed no significant impact on the tumor growth rate. Despite this, when stable cells were injected into the tail veins of nude mice, the increased lung metastases induced by mutant p53 G245D expression were significantly suppressed by overexpression of FOXO3a (Fig. 7a, b, Supplementary Fig. 7a). Similarly, down-regulation of FOXM1 in mutant p53 UM-SCC-1 cells also impaired the development of mutant p53-mediated lung metastases (Fig. 7c, d, Supplementary Fig. 7b). In further support of the regulatory role of mutant p53 G245D in FOXO3a-FOXM1 signaling, IHC staining of lung metastatic tumor lesions also showed that while mutant p53 G245D decreased FOXO3a’s (Fig. 7e, f) but increased FOXM1’s (Fig. 7g, h) nuclear staining, further overexpression of FOXO3a in mutant p53 UM-SCC-1 cells (Fig. 7e) decreased the mutant p53-induced FOXOM1 nuclear staining (Fig. 7g, h). Altogether our results suggest that while mutant p53 G245D can modulate FOXO3a-FOXM1 signaling in vivo, both FOXO3a and FOXM1 can regulate GOF mutant p53-mediated pulmonary metastasis of UM-SCC-1 cells in nude mice.

### HNSCC patients with *TP53* mutations and high levels of *FOXM1* expression have the poorest survival outcomes

Finally, given our results showing regulation of *FOXM1* by GOF mutant p53, we analyzed the FOXM1 RNAseq expression data from The Cancer Genome Atlas HNSCC data set. As shown in Fig. 7i, oral cancer patients with *TP53* mutations (*n* = 232) had a significantly higher mean level of *FOXM1* mRNA expression than patients with wild-type *TP53* (*n* = 75). Moreover, HNSCC patients with both *TP53* mutations and high levels of *FOXM1* expression (*n* = 177) had lower survival rates than the patients with wild-type *TP53* (*n* = 151) or with mutant *TP53* and low levels of *FOXM1* expression (*n* = 177) (Fig. 7j). This not only suggests the potential prognostic value of *TP53* mutations and *FOXM1* expression but also highlights their important roles in tumorigenesis and progression of HNSCCs.

## Discussion

In this study, we have shown that the expression of GOF mutant p53s can up-regulate *FOXM1* expression in HNSCC cells. Our results also indicate that GOF mutant p53s inhibit the function of FOXO3a through suppression of its nuclear localization and phosphorylation mediated by AMPK. Moreover, we have demonstrated that overexpression of *FOXO3a* decreases but down-regulation of *FOXO3a* increases GOF mutant p53-mediated *FOXM1* expression, further supporting the hypothesis that FOXO3a is a negative regulator of *FOXM1*. Our results revealed that either overexpression of *FOXO3a* or down-regulation of *FOXM1* impairs GOF mutant p53-mediated cell invasion in vitro and pulmonary metastases of UM-SCC-1 cells in vivo. Finally, we have shown that HNSCC patients with *TP53* mutations and high levels of *FOXM1* expression have poorer survival outcomes than those without one or both of these features, indicating the potential prognostic value of *TP53* mutations and FOXM1 expression in HNSCC. Together with our previous observation that GOF mutant p53s inhibit AMPK [16], our current study strongly suggests that GOF mutant p53s can activate *FOXM1* through inhibiting AMPK and its downstream target FOXO3a (Fig. 7k) and that the mutant p53-AMPK-FOXO3a-FOXM1 signaling axis is one of mechanisms through which mutant p53s gain oncogenic activity in HNSCC.

Thus far, most proposed GOF mechanisms of mutant p53s have involved transcription-dependent gene regulation, and many GOF mutant p53s have been shown to directly interact with transcription factors and cofactors, thereby modulating gene transcription [8, 10, 11]. Depending on the cell context, several transcription factors (e.g., ER, E2F, FOXO, and YAP1) have been shown to regulate *FOXM1* transcription (Fig. 4a) [19, 34]. Notably, some GOF mutant p53s were reported to interact with transcription factors E2F1 and YAP1 as well [35, 36]. This raises the possibility that GOF mutant p53s G245D and C238F might also be able to regulate *FOXM1* expression through directly targeting these transcription factors in UM-SCC-1 cells. While these possibilities still need to be tested, our results here nevertheless provide us an excellent example of transcription-independent regulation (i.e., inhibition of phosphorylation/activation of AMPK and FOXO3a) of mutant p53 GOF. More importantly, such transcription-independent regulation can eventually lead to transcription-dependent regulation of downstream targets (e.g., *FOXM1*), thereby contributing to GOF activities of mutant p53s.

Previously, *FOXO3a* was shown to cooperate with wild-type p53 signaling. Not only does FOXO3a interact with, stabilize, and share some common target genes with wild-type p53 [37–41], but also wild-type p53 is a direct transcription activator of *FOXO3a* [42], which is consistent with FOXO3a’s tumor suppressive role. Likewise, FOXM1 is also involved in wild-type p53 signaling. However, instead of being activated by wild-type p53 like FOXO3a, *FOXM1* expression has been shown to be suppressed by wild-type p53 indirectly, through p21- and pRB-dependent mechanisms [43, 44]. In support of FOXM1’s oncogenic role, our results also showed that FOXM1 expression is inhibited by wild-type p53 (Fig. 2i). This constellation of findings helps to explain why oral cancer patients who had tumors with wild-type p53 also had a lower mean level of *FOXM1* expression than those who had tumors with mutant p53s (Fig. 7i), in which merely the loss of wild-type p53’s function due to mutation would be expected to relieve wild-type p53-mediated repression of *FOXM1*. Moreover, our results presented here strongly suggest that some GOF p53 mutants not only lose wild-type p53-mediated *FOXM1* suppression but can actually acquire the ability to promote *FOXM1* expression through suppression of FOXO3a’s function (Fig. 7k). Therefore, while wild-type p53 and FOXO3a form a mutual regulatory network that plays an important role in their tumor suppressive functions, certain GOF mutant p53s acquire the ability to modulate FOXO3a-FOXM1 signaling in a way contrary to wild-type p53. Instead of stimulating *FOXO3a* and inhibiting *FOXM1* like wild-type p53, GOF mutant p53s can suppress FOXO3a function and promote *FOXM1* expression, increasing the cell invasion and metastatic potentials of HNSCC cells. This observation could help to explain why HNSCC patients with both *TP53* mutations and high levels of *FOXM1* expression had the poorest survival outcomes among those we studied (Fig. 7j), and this knowledge will help us not only understand the molecular pathogenesis of HNSCCs but also develop therapeutic strategies against them.

HNSCC arises from a multistep process of carcinogenesis, in which *TP53* mutations often occur early and can be detected in premalignant lesions [4]. Consistent with this, *FOXM1* has been shown to be significantly upregulated in both human oral premalignant and HNSCC tissues [45], suggesting that upregulation of *FOXM1* is also an early event in HNSCC development. The correlation of both *TP53* mutations and *FOXM1* upregulation in an early stage of HNSCC development strongly indicates that the interplay of *TP53* mutations and *FOXM1* are pathologically relevant, in which both loss of *FOXM1* repression due to p53 mutations and activation of *FOXM1* expression by mutant p53 GOF play important roles contributing to HNSCC development and progression. Finally, our results indicate that neither FOXO3a nor FOXM1 influences the primary tumor growth of UM-SCC-1 cells expressing GOF mutant p53 in vivo, but both affect the cell invasion and metastasis of HNSCC cells that carry GOF mutant p53s. Since metastases of HNSCC are associated with poor survival of patients with HNSCC, understanding signaling pathways associated with metastases such as the mutant p53-AMPK-FOXO3a-FOXM1 pathway will offer us the opportunity to develop therapeutic strategies for HNSCC patients.

In summary, we have demonstrated in both in vitro cell cultures and in vivo tumor models of HNSCC that mutant p53s can acquire GOF properties through a transcription independent mechanism promoting *FOXM1* expression through inhibition of AMPK-mediated FOXO3a activation. Targeting the GOF mutant p53-AMPK-FOXO3a-FOXM1 axis could be an effective therapeutic approach for treating HNSCC and for improving the survival of patients with HNSCC.

## Materials and methods

### Cell lines and DNA

The STR-verified HNSCC cell lines UM-SCC-1, UMSCC-17B, Detroit 562, PCI-13, MDA1586, PCI-15B, TR146, and Ca9-22 were described previously [46], and STAR cells was established in our laboratory. Cells were cultured in Dulbecco’s modified Eagle’s medium supplemented with 10% FBS. Stable cell lines expressing control vector pBabe; wild-type p53; mutant p53s G245D, C238F, R175H, and E336X; and short hairpin RNAs (shRNAs) for p53 (shp53) and AMPKα1 (shAMPK α1) were established as described previously [9, 16]. pBMN-GFP (Addgene, Cambridge, MA, USA), pLVX-IRES-mCherry (Takara Bio USA, Mountain View, CA, USA), and the PCR product from pcDNA3-FLAG-FKHRL1 (Addgene) were used to generate retroviral pBMN-GFP-FOXO3a and lentiviral pLVX-IRES-mCherry-FOXO3a expression. Lentiviral pGIPz shRNA constructs shFOXO3A-A (V3LHS_375381), shFOXO3A-B (V3LHS_375386), shFOXM1-A (V2LHS_283849), shFOXM1-C (V3LHS_396939), shp53-A (V3LHS_333920), and shp53-C (V3LHS_333919) were from Dharmacon (Lafayette, CO, USA). *AMPKα1* and *FOXO3a* CRISPR/Cas9 knockout (KO) plasmids sc-400104 and sc-400308 were from Santa Cruz Technologies (Dallas, TX, USA).

### mRNA expression array and the cancer genome atlas data analyses

Total RNAs were used for Affymetrix GeneChip® Human Gene 2.0 ST Array. Using the aroma.affymetrix package in R, we quantified the CEL files with Robust Multi-Array Average (RMA) background correction, quantile normalization, and RmaPlm (Probe-Level Models using RMA) summarization. After processing the data, log2 transformation was applied. Two-sample *t*-test was used for identification of differentially expressed genes. *P*-values from two-sample t-tests were modeled by a beta-uniform mixture model, and significance cutoff was determined by false discovery rate (0.01). Normalized RNAseq data from 505 TCGA HNSCC samples (http://gdac.broadinstitute.org/) was used for *FOXM1* expression analysis.

### Immunoblotting and immunohistochemistry antibodies

The primary antibodies used for immunoblotting were: FOXM1 (Cell Signaling, Beverly, MA, USA; 5436), phospho-FOXO3a S413 (Cell Signaling; 8174), FOXO3a (Cell Signaling; 12829), phospho-AMPKα1 T172 (Cell Signaling; 2535), AMPKα1 (Cell Signaling; 2795), AMPKα (Cell Signaling; 2603), phospho-AKT S473 (Cell Signaling; 4051), AKT (Cell Signaling; 9272), p53 (Santa Cruz; sc-126), and β-actin (Sigma-Aldrich, St. Louis, MO, USA; A5316).

### Quantitative reverse transcription PCR analyses

Quantitative reverse transcription PCR (RT-qPCR) was performed with Power SYBR Green PCR Master Mix (ThermoFisher Scentific, Waltham, MA, USA), using the following primers: FOXM1, 5′-ACGTCCCCAAGCCA GGCTC-3′ (forward) and 5′-CTACTGTAGCTCAGGAATAA-3′ (reverse); FOXM1b, 5′-CCAGGTGTTTAA GCAGCAGA-3′ (forward) and 5′-TCCTCAGCTAGCAGCACC TTG-3′ (reverse); FOXM1c, 5′-CAATTGCCCGA GCACTTGGAATCA-3′ (forward) and 5′-TCCTCAGCTAG CAGCACCTTG-3′ (reverse); and TBP, 5′- GCACAGGAGCCAAGAGTGAA -3′ (forward) and 5′-TCAC AGCTCCCCACCATGTT -3′ (reverse). The TBP gene was used as an internal control. The fold-change in expression (*n* = 3/group) and standard deviation were then calculated using the comparative CT method.

### In vitro transwell invasion assay

In total 5 × 10^4^ cells were resuspended in serum-free and glucose-free DMEM and seeded into the interior of BD BioCoat Matrigel Invasion inserts (*n* = 3/group). Once the cells were seeded, DMEM supplemented with 20% FBS was placed into the lower well as the chemoattractant. After 22 h incubation, non-invasive cells were removed from the upper surface, and the remaining cells were stained with crystal violet or the Hemacolor stain set (EMD Millipore, Burlington, MA, USA) and then quantified.

### 3D cell culture

A 100 μl of collagen gel (Nitta Gelatin, Osaka, Japan) was layered on the bottom of 48-well for 30 min to allow the collagen gel to solidify. In total 3000 cells in 150 μl of collagen gel (*n* = 3/group) were then layered on top of the solidified collagen gel. When all the collagen gel was solidified, 1 ml of DMEM containing 10% FBS was added.

### Animal tumor models

All experiments involving mice were approved by MD Anderson Cancer Center Institutional Animal Care Regulations and Use Committee. For orthotopic nude mouse oral cancer model, stable cells were injected into the dorsal part of tongues of 6- to 8-week-old male athymic nude mice (*n* = 10/group, randomized according to body weight); the injections consisted of 5 × 10^4^ cells suspended in 30 μl of serum-free DMEM, as described previously [47]. For lung metastatic model, UM-SCC-1 stable cells were injected into tail vein of 6- to 8-week-old male athymic nude mice (*n* = 10/group, randomized according to body weight). The injections consisted of 5 × 10^5^ cells suspended in 200 μl of PBS, as described previously [9]. The mice died within 1 week after the injection were excluded from the analyses. After mice were killed, lungs were collected, embedded, sectioned, stained, and scanned by Aperio AT2 (Leica, Wetzlar, Germany). The whole lung area and metastatic area were calculated by ImageJ software to analyze metastatic ratio.

### Statistical analysis

Two-Way ANOVA (mouse studies), one-way ANOVA (Transwell invasion assay), the student *t*-test (evaluation of lung metastases, 3D culture and RT-qPCR), and the chi-square test (immunohistochemical analyses) were used to compare samples between the control and test groups.

## Electronic supplementary material

Supplemental Figures S1-S7

Supplemental Table S1
