# Supplementary material for: Gain-of-function mutant p53 promotes the oncogenic potential of head and neck squamous cell carcinoma cells by targeting the transcription factors FOXO3a and FOXM1
Source: Oncogene. 2017 Dec 22;37(10):1279–92. doi: 10.1038/s41388-017-0032-z (PMC5844800; doi:10.1038/s41388-017-0032-z)
Supplement: Supplementary file 1 — Supplemental Figures S1-S7 [file 41388_2017_32_MOESM1_ESM.pdf]

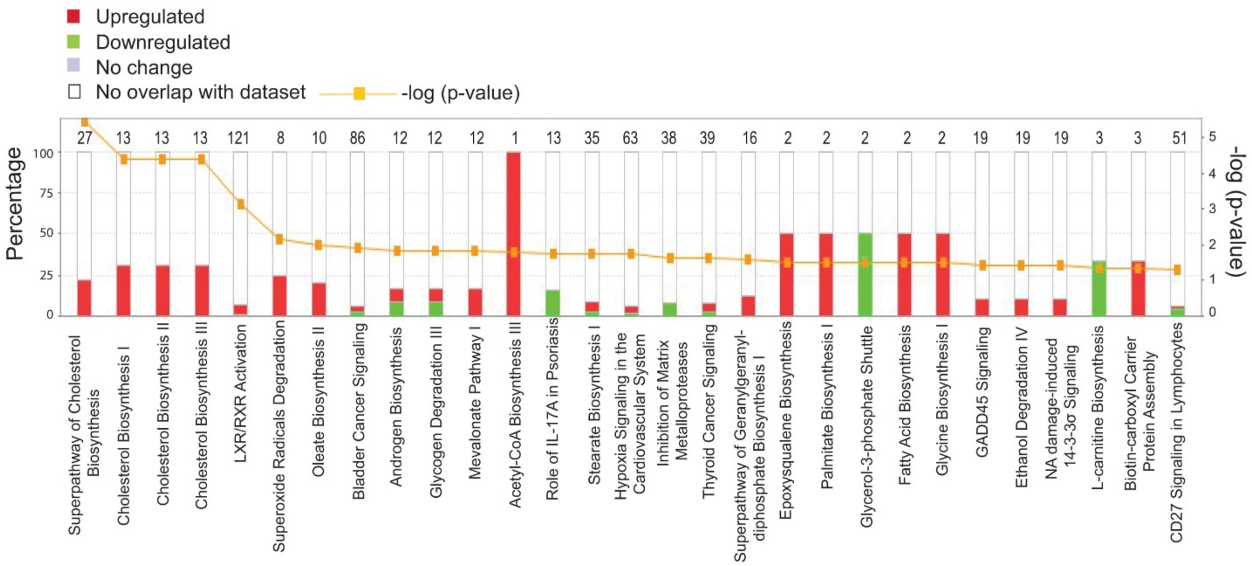

Figure S1: Ingenuity Pathway Analysis of the top signaling pathways in which 91 differentially expressed genes between pBabe and mutp53 G245D UM-SCC-1 cells.

A

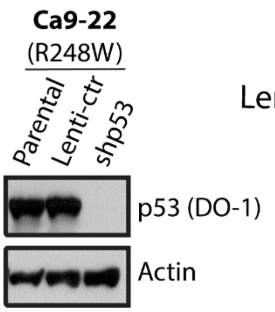

B

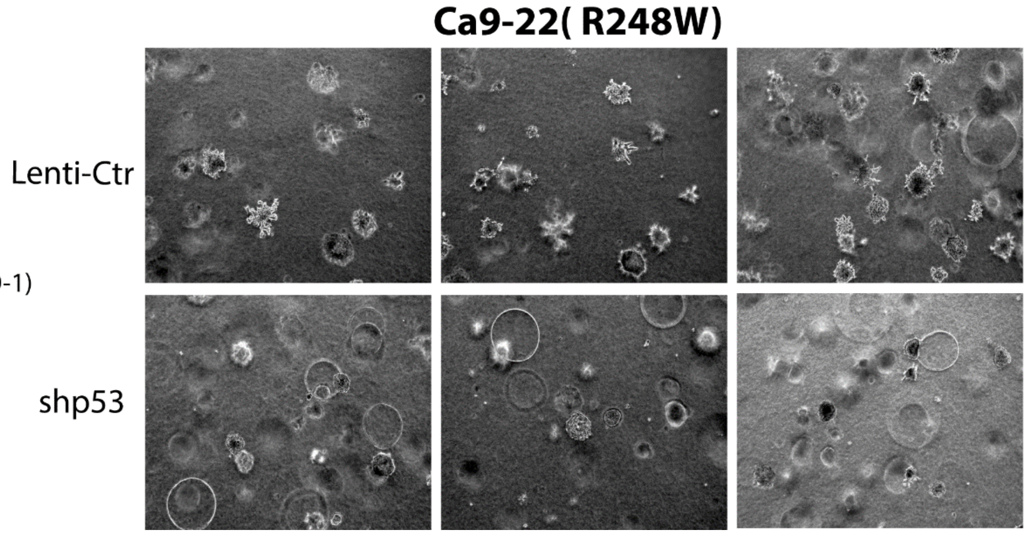

C

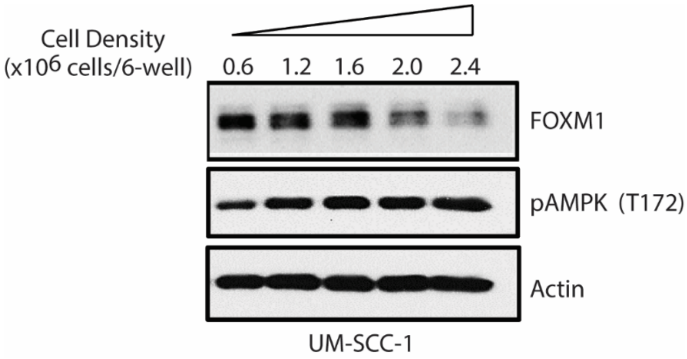

Figure S2: (A) Western blot of Ca9-22 control and shp53 cell lines. (B) Ca9-22 control and shp53 cell lines 8 days after collagen gel 3D culture. In each group, three representative images were taken. (C) Expression of FOXM1 in a cell-density-dependent manner. Western blot analysis of UM-SCC-1 cells under different cell-density culture conditions in 6-well plates.

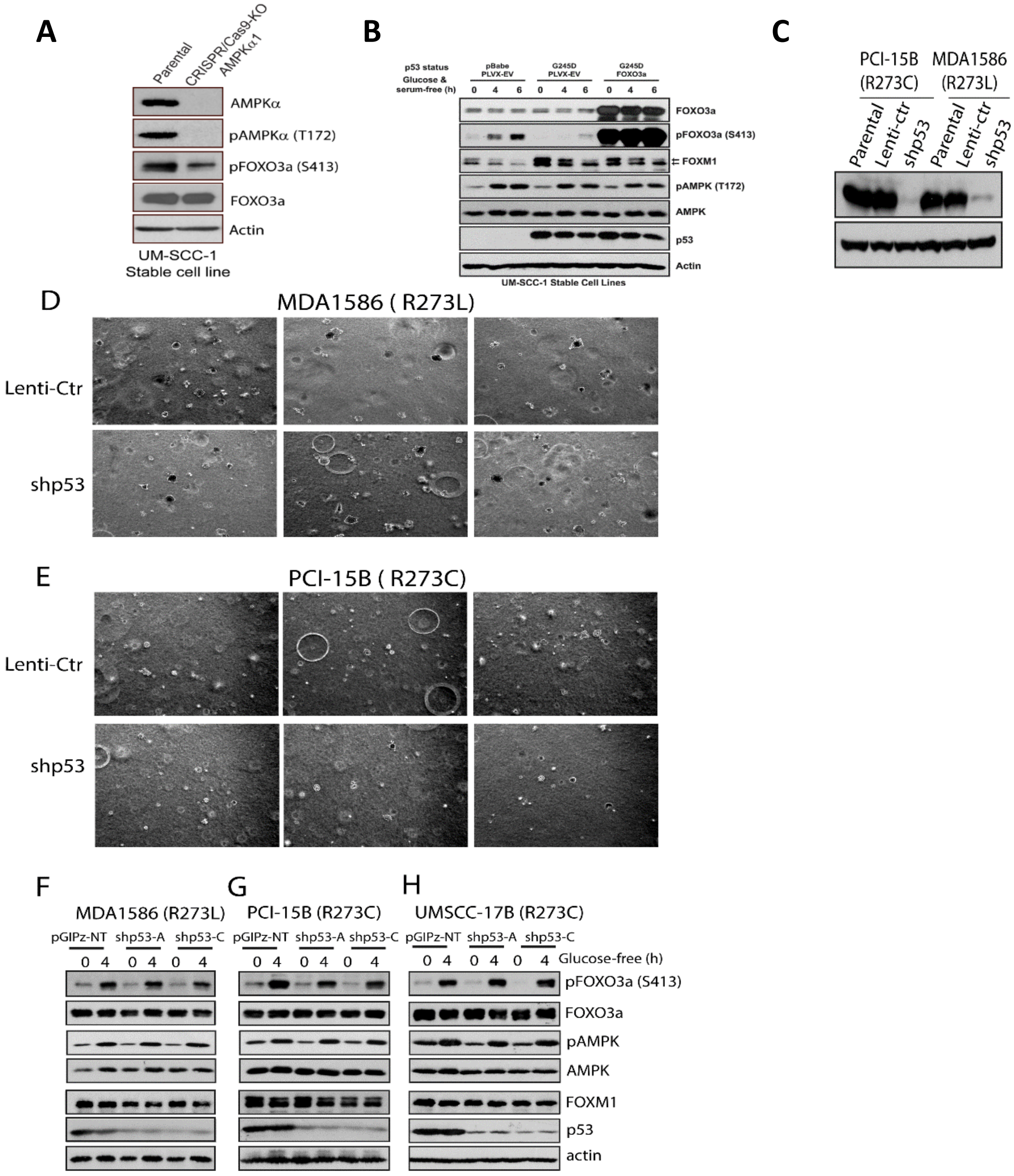

Figure S3: (A-C) (F-H) Western blot analyses of HNSCC stable cell lines. (D-E) MDA1586 and PCI-15B control and shp53 cell lines 8 days after collagen gel 3D culture. In each group, three representative images were taken.

A

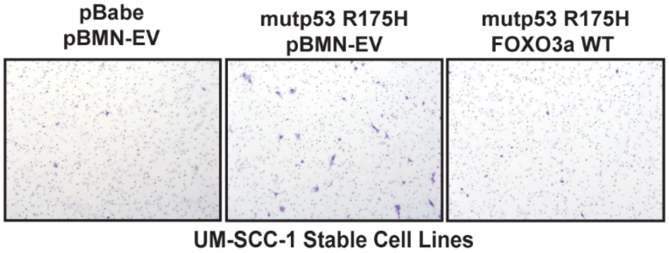

B

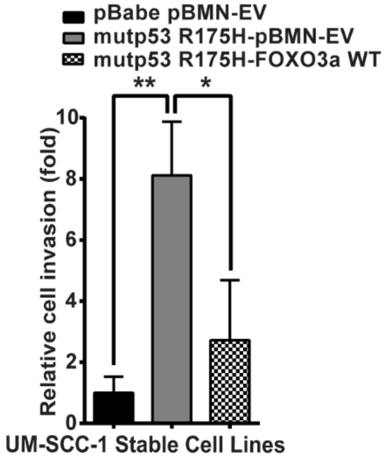

Figure S4: (A) Representative images of Transwell invasion assay of UM-SCC-1 stable cell lines. (B) Quantitation of invasive cells in (A). *, $p<0.05$; **, $p<0.01$.

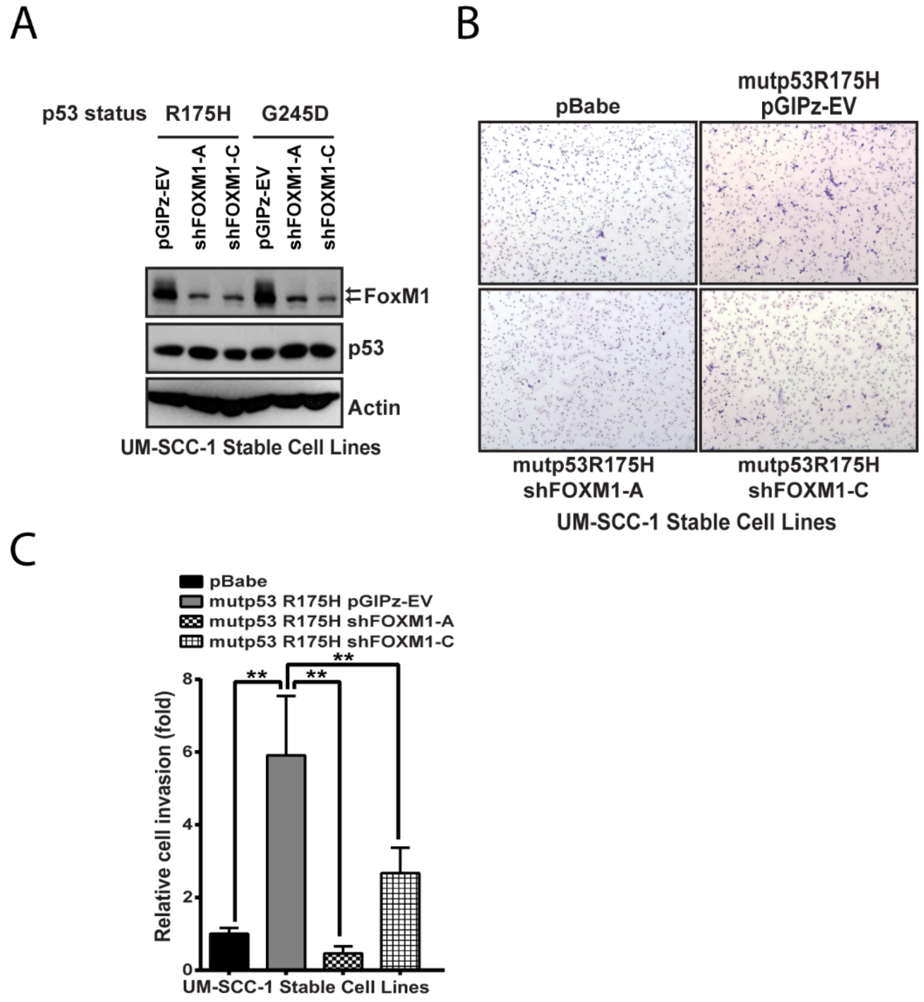

Figure S5: (A) Western blot analysis of isogenic UM-SCC-1 stable cell lines. (B) Representative images of Transwell invasion assay of UM-SCC-1 stable cell lines. (C) Quantitation of invasive cells in (B). **, $p<0.01$.

A

B

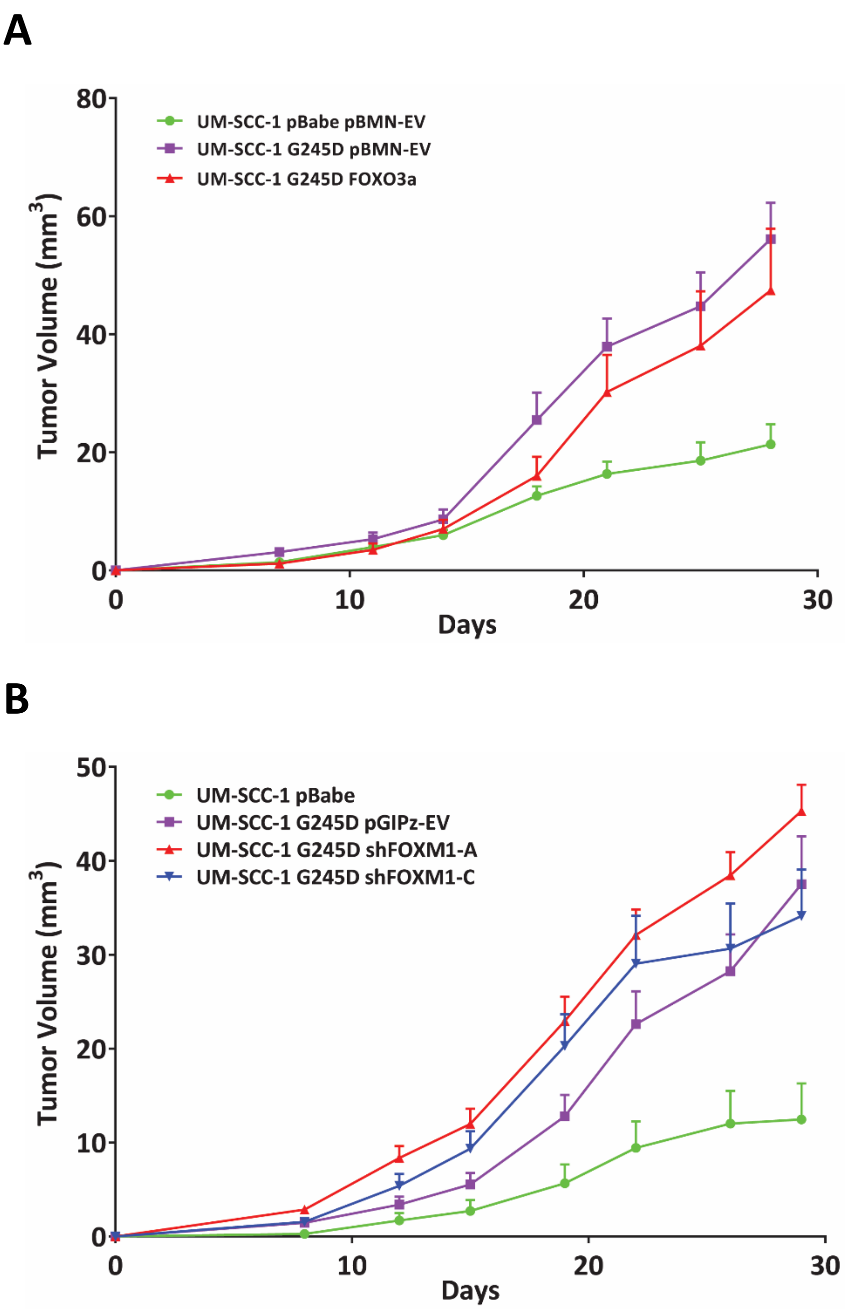

Figure S6: Growth of tumors from UM-SCC-1 stable cell lines injected into the tongues of nude mice.

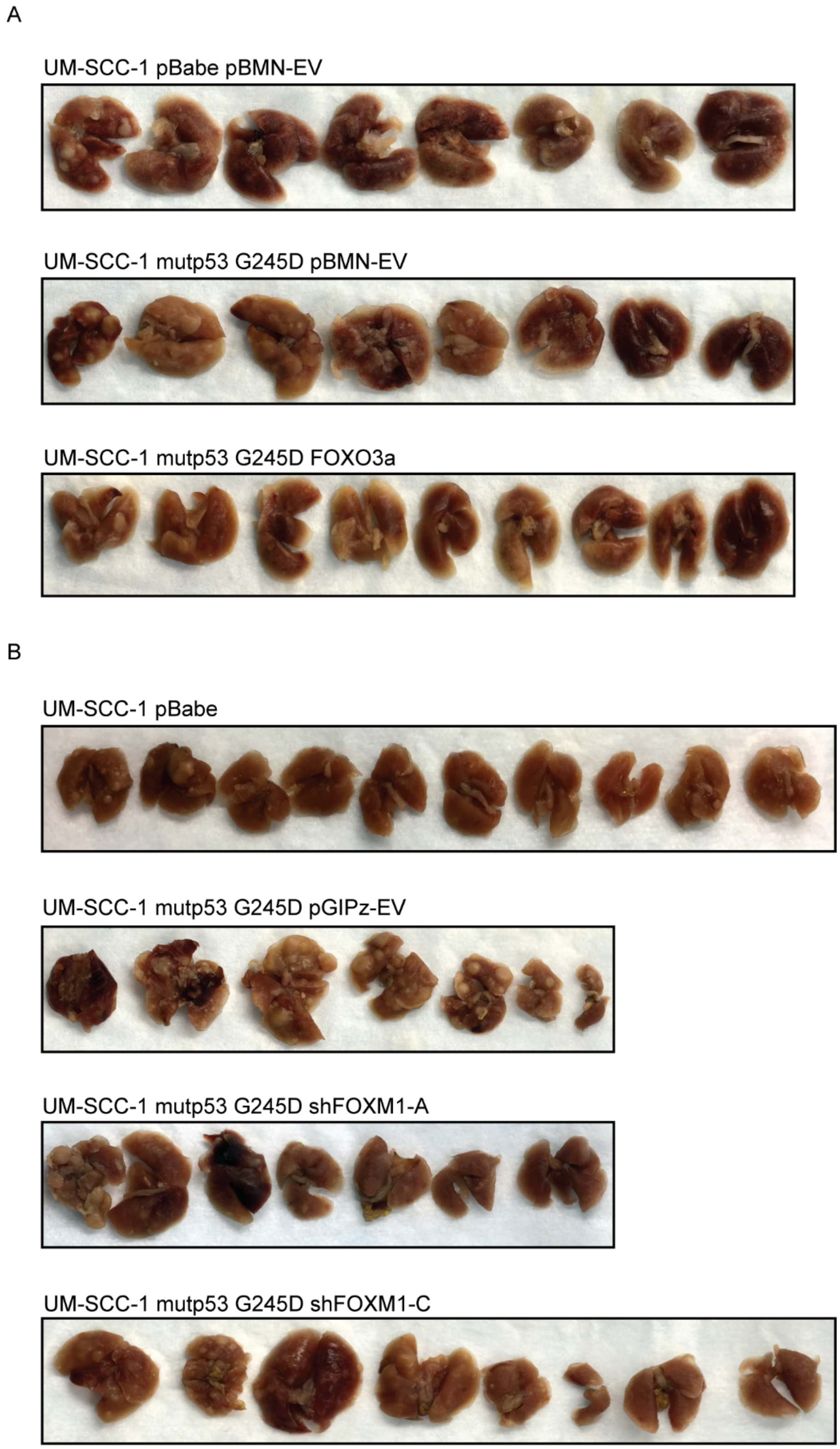

Figure S7: Lungs harvested after injection of tumor cells into the tail veins. (A) Lungs harvested 13 weeks after FOXO3a stable cell line injection; (B) Lungs harvested 14 weeks after shFOXM1 stable cell lines' injection.
